# The human CD40 agonistic antibody ADC-1013 generates immune mediated anti-tumor effects in syngeneic tumor models in hCD40 transgenic mice

**DOI:** 10.1186/2051-1426-2-S3-P251

**Published:** 2014-11-06

**Authors:** Sara M Mangsbo, Niina Veitonmäki, Erika Gustfson, Karin Enell Smith, Christina Furebring, Per Norlén, Malin Lindstedt, Thomas H Tötterman, Peter Ellmark

**Affiliations:** 1 Immunology, Genetics and Pathology, IGP, Uppsala University, Uppsala, Sweden; 2 Alligator Bioscience AB, Lund, Sweden; 3 Immunotechnology, Lund University, Sweden

## 

Local activation of costimulatory pathways by e.g. CD40 activation has been shown to generate powerful systemic anti-tumor responses. Here we report significant anti-tumor effects obtained with an optimized fully human agonistic CD40 antibody, ADC-1013, in two syngeneic tumor models.

An hCD40 transgenic mouse (hCD40tg) strain was used to evaluate the immune mediated anti-tumor effects of ADC-1013. Dendritic cells obtained from hCD40tg mice were hCD40 positive and could be activated by stimulation with ADC-1013 to a similar extent as human monocyte derived dendritic cells. Furthermore, stimulation of dendritic cells from hCD40tg mice *in vitro*, with ADC-1013, induced antigen specific T cell activation.

Two different syngeneic tumor models, hCD40 negative MB49 bladder cancer and hCD40 positive B16.F10.hCD40+ melanoma, was used to demonstrate anti-tumor effects. Subcutaneous tumors from both models were characterized by flow cytometry and immunohistochemistry in hCD40tg mice. Treatment of the bladder cancer model (MB49) with ADC-1013 resulted in significant anti-tumor response and long term tumor immunity. The anti-tumor immunity was shown to be T cell dependent and naïve mice were protected from tumor challenge by transplantation of splenocytes from cured hCD40tg mice. In addition, significant anti-tumor effect was demonstrated in a subcutaneous B16.F10.hCD40+ melanoma model.

The human CD40 agonistic antibody ADC-1013 is the first costimulatory antibody optimized for local immunotherapy of cancer. Strong immune mediated anti-tumor effects were demonstrated. ADC-1013 is currently in late pre-clinical development.

